# Evaluation of dentifrices of complementary and alternative medicinal systems on plaque formation and gingivitis: A randomized controlled clinical trial

**DOI:** 10.4317/jced.56333

**Published:** 2020-03-01

**Authors:** Manumanthu-Venkata Ramesh, Puvvadi-Gopala-Krishna-Naveen Kumar, CR Allamaprabhu, Nerella-Narendra Kumar, Syed-Amtu Yasmeen, Gadde Praveen, Thorreti-Venkata-Sandhya Lakshmi

**Affiliations:** 1 Reader, Department of Public Health Dentistry, Vishnu Dental College, Bhimavaram – 534202, Andhra Pradesh, India; 2 Professor, Department of Public Health Dentistry, Faculty of Dental Sciences, IMS, BHU, Varanasi, Uttar Pradesh, India; 3 Reader, Department of Public Health Dentistry, College of Dental Sciences, Davangere, India; 4 Department of Oral Medicine and Radiology, Narayana Dental College and Hospital, Nellore, India; 5 Senior Lecturer, Department of Oral Medicine and Radiology, Vishnu Dental College, Bhimavaram, India; 6 Reader, Department of Public Health Dentistry, Vishnu Dental College, Bhimavaram, India; 7 Department of Prosthodontics, MNR Dental College and Hospital, Sangareddy, India

## Abstract

**Background:**

Bio-film formation is a natural process in the oral environment, but needs to be controlled through regular brushing in order to prevent the development of caries and periodontal diseases.

**Material and Methods:**

A wide variety of dentifrices of complementary and alternative medicinal systems are available in the market which claims superior plaque control. A randomized controlled double-blinded concurrent parallel clinical trial was conducted with the objective to evaluate and compare the clinical effectiveness of three commercially available dentifrices of complementary and alternative medicine systems with that of a placebo dentifrice on Gingival Index (GI) and Plaque Index (PI) scores after 15, 30, 45 and 60 days of usage among 80 adults aged 35-44 years.

**Results:**

The mean GI scores reduced by 29.19 %, 22.28 %, 32.43 % and 10.33 % in the herbal dentifrice, homeopathic dentifrice, conventional dentifrice, and placebo dentifrice groups by the end of the study period. Highest reduction of 33.5% and 34.87 % in PI scores were seen in the herbal and conventional dentifrice. This reduction was statistically significant (*p*=0.001).

**Conclusions:**

The herbal dentifrice tested in the present study has demonstrated anti-gingivitis and anti-plaque efficiency equivocal to the conventional dentifrice. The homeopathic dentifrice was as good as a placebo.

** Key words:**CAM dentifrices, Homeopathic dentifrice, plaque, gingivitis, tooth brushing.

## Introduction

World Health Organization in 2010 declared that “Oral health is essential to general health and quality of life. It is also a basic human right” (1). Dental caries and periodontal disease, the most common diseases afflicting the oral cavity, have been responsible for untold pain, suffering, tissue destruction, tooth loss. These diseases being infectious in nature appear consequent to dental plaque bio-film accumulation. The 1998 European Workshop on Mechanical Plaque Control emphasized the importance of regular oral hygiene practices in effective removal of dental plaque to enhance dental and periodontal health throughout life. At present, mechanical cleaning using a toothbrush and a dentifrice is the most widely used method of plaque control (2).

In a constant quest to enhance oral health, a number of synthetic chemical agents with potential adjunctive benefits were added to the existing dentifrices. Most of these agents displayed good anti-plaque activities. Concurrently, side effects like teeth staining and taste alterations too were reported on long-term usage (3). To overcome the drawbacks of synthetic chemicals, the expedition for alternate natural products began. Concomitantly, in search for solace in traditional or indigenous medicine, civic awareness with regard to natural products is on the rise. Traditional medicine is becoming popular owing to factors such as availability, affordability, biocompatibility, cultural familiarity and family influence. In light of these facts, the health care approach is directed towards other holistic methods for managing diseases and conditions and dentistry is not an exception (4).

Ayurveda, one of the oldest systems of medicine from India, is nearly 5000 years old. Ayurvedic herbs have nature’s own power of remedies. Herbal tooth powder and toothpaste containing active extracts of botanicals such as Neem, Clove, Fennel, Meswak and other plants are formulated to promote healthy gums and teeth. Manufacturers of these herbal kinds of toothpaste also claim to avoid common “unnatural” ingredients and offer a high margin of safety (5).

Homeopathy, discovered by German Physician Samuel Hahnemann has been in existence for over 200 years. Homeopathic remedies are mostly derived from plants, but also minerals, animal products, healthy and unhealthy tissues, and secretions and other sources. It has emerged as one of the alternative therapy in cases of treatment failure or poor response to conventional drugs. Many homeopathic remedies like Antimonium crudum, Calcarea carbonica and Chamomille have been found to be effective for oral conditions too (6).

The ultimate goal of medicine, regardless of the type of health system, is to benefit the patient. Undeniably, we cannot do this without the foggiest thought on what each system of medicine has in the offering relative to the other. Also, as comparative studies related to dentifrices of the aforementioned Complementary and Alternate Medicine systems (CAM) are lacking, we encounter a piquant situation when patients seek our opinion on such oral health care products. Research in this area to generate the necessary evidence, is required. Thus, to evaluate and compare the effects of commercially available herbal, homeopathic, conventional and a formulated placebo dentifrice on plaque accumulation and gingivitis, this randomized controlled doubled blinded clinical trial was conducted among subjects aged 35-44 years in Davangere city, Karnataka.

## Material and Methods

The proposal for this randomized controlled double-blinded (observer and subject) parallel-group clinical trial was submitted for approval and ethical clearance was obtained from the Institutional review board of College of Dental Sciences, Davangere. This trial protocol is registered with the Clinical Trials Registry of India bearing the registration number CTRI/2018/04/013103. A pilot study was conducted to check the feasibility and validity of the study. Pilot study assessments were utilized for proper planning and execution of the main study. Twelve subjects who participated in the pilot study were not included in the main study. At a level of significance set at 5%, power at 80% and for an expected effect size of 0.44 (obtained from the pilot study), it was determined that 16 subjects per group were required to perform the study. However to compensate for dropouts, if any, a sample size of minimum 20 subjects in each group was used thus totaling to an effective sample size of 80.

The study subjects were the teaching and non-teaching faculty of ARG College of Arts and Commerce, Davangere. 80 eligible subjects that fulfilled the inclusion criteria were recruited. Adults aged 35 to 44 years with good general health, who are willing to participate and provide informed consent having a minimum of 20 natural permanent teeth and with a Gingival Index score ≥ 1 are included. Participants with known hypersensitivity to the products used in the study and unable to comply with the study appointment schedules, having oral soft tissue pathologies, advanced periodontal disease (probing depth > 3mm) or wearing an intraoral appliance/dentures, those under antimicrobial therapy at least 1 month prior to the study, smokers and pregnant women were excluded.

The subjects were randomly allocated into four groups using the lottery method with 20 subjects in each group. The assignment was performed by a person not involved in the examination.

The four intervention groups are 

Group I – Herbal based commercial dentifrice (Himalaya Complete care, a product of Himalaya Herbals, Bangalore, India)

Group II - Homeopathic based commercial dentifrice (Homeodent, a product of SBL, Jaipur, India)

Group III - Conventional commercial dentifrice (Colgate Total, a product of Colgate-Palmolive India Ltd)

Group IV - Prepared placebo dentifrice

**Figure 1:**
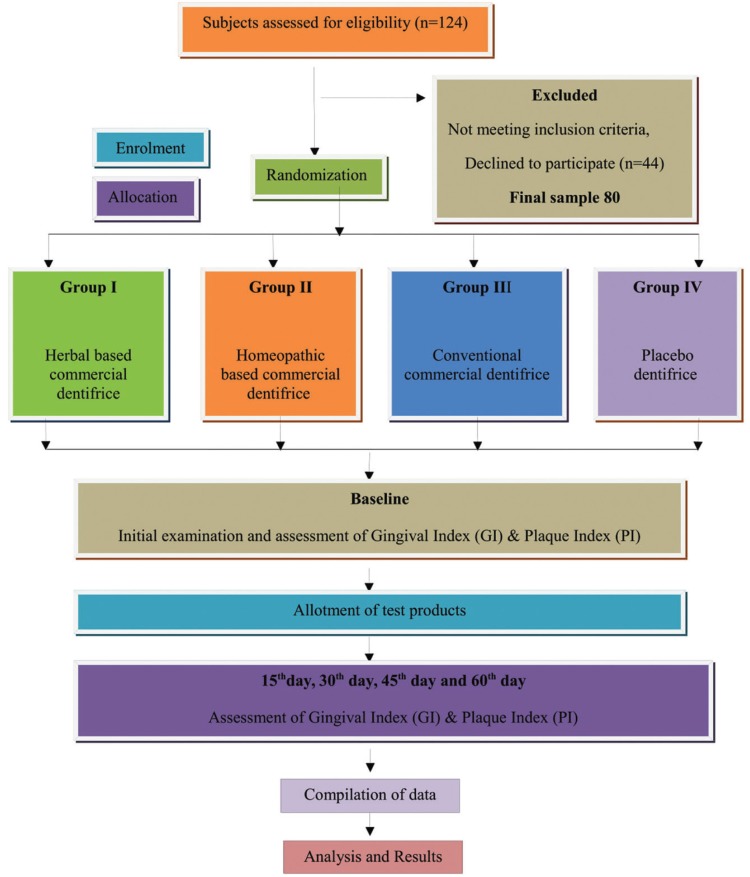
Schematic Representation of Study Design.

Commercially available dentifrices used in the study, i.e. herbal dentifrice, homeopathic dentifrice, and conventional dentifrice were obtained from a local market. The placebo dentifrice was manufactured at Department of Pharmacognosy, Bapuji College of Pharmacy, Davangere. Commercially available dentifrices were chosen after studying their composition and the placebo dentifrice was formulated such that it differed from the other test dentifrices only in terms of the active ingredient i.e., herbal, homeopathic and conventional components added by the manufacturer. Placebo dentifrice was used as a reference unit to assess the therapeutic benefit of active ingredients claimed by the manufacturers. All the dentifrices were transferred to identical laminate tubes each having a capacity of 10 grams so as to blind the subjects as to which dentifrice they are using. The tubes were then number coded by an assistant, who then administered it, thus ensuring double blinding i.e. blinding of subjects and the principal investigator. The entire study protocol was presented in Figure 1.

Study participants were instructed to brush their teeth with their regular brush, but without toothpaste for a period of 1 week before the baseline examination. This washout period was maintained to eliminate the residual carry-over effects of the dentifrices used previously by the subjects. WHO Type III examination of all the study subjects was done at baseline (T1), on 15th day (T2), 30th day (T3), 45th day (T4) and 60th day (T5) after 59 days of intervention for recording Modified Plaque Index (Turesky S., Gilmore N.D. and Glickman I. 1970) (7) and Gingival Index (Loe H. and Silness J. 1963) (8) by a single examiner. As single examiner was involved in recording the indices, intra-examiner calibration was done. A high kappa co-efficient value of 0.86 and 0.84 respectively for modified plaque index and gingival index respectively reflected a high degree of conformity in observational judgments of the examiner.

Participants were provided with the test dentifrices and were asked to use these dentifrices twice daily before breakfast and bedtime throughout the study period. They were asked to abstain from dentifrices other than those provided for the study and to maintain their normal dietary habits throughout the study. The subjects were instructed to wet the brush and squeeze about 1.5 ml of toothpaste over the bristles and to spread it evenly and brush their teeth in their usual fashion. They were told not to expectorate during brushing. After brushing the teeth, they were asked to swish the dentifrice foam for 1 min before expectorating. All the subjects were instructed to maintain a chart on daily product use. After 15 days, the first follow up examination was performed and compliance with the study regimen and appearance of side effects were assessed. Additional supplies of dentifrices were provided at each follow-up visit.

Data collected in the present study was compiled in Microsoft Excel sheet and analyzed using the Statistical Package for Social Sciences (SPSS) version 20.0. The variation in mean PI and GI scores between different groups at same time interval was analyzed using one-way Analysis of Variance (ANOVA) followed by Tukey’s Post hoc test and for within group at different time intervals was analyzed using Repeated measures ANOVA followed by pairwise comparisons using LSD Bonferroni test. *p*-value ≤ 0.05 was considered statistically significant.

## Results

A total of 80 subjects (48 males and 32 females) were included in the present study. Table 1 depicts the product wise distribution of study subjects according to gender. The mean age of the subjects was 38.50 (± 2.42) years. All the 80 subjects displayed compliance with the study protocol and there were no dropouts. None of the study participants reported side effects on dentifrice usage.

**Table 1:**
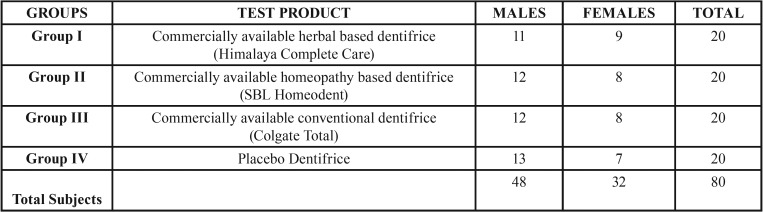
Distribution of study subjects according to Gender.

**Figure 2:**
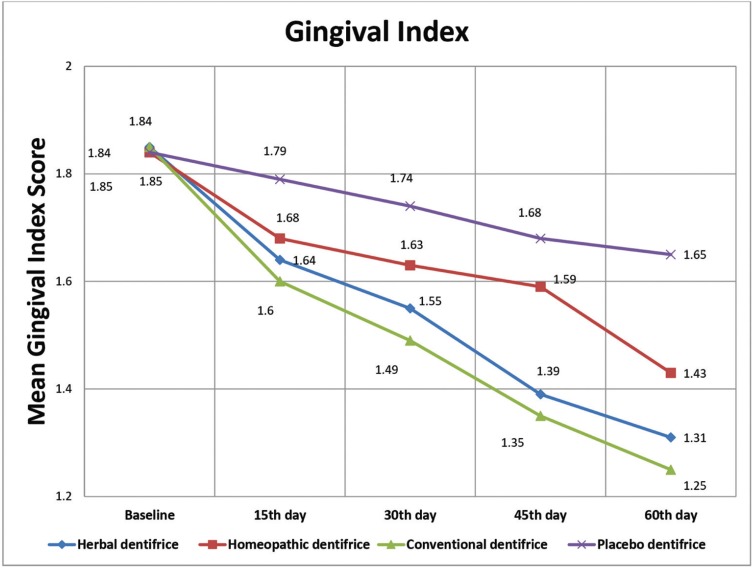
Comparison of the dentifrice groups with respect to mean Gingival Index scores at various time intervals.

Table 2 and Figure 2 outline the inter-group and intra-group comparison of mean GI scores. The mean Gingival Index (GI) scores in the herbal (Group I), homeopathic (Group II), conventional (Group III) and placebo dentifrice (Group IV) groups were 1.85 ±0.22, 1.84 ±0.22, 1.85 ±0.20 and 1.84 ±0.24 respectively at baseline (T1). There was no statistically significant difference between the groups (*p*=0.995). The statistical difference between the groups was found non-significant (*p*= 0.210) even at the end of 15 days (T2). At the end of 30 days (T3), the mean GI score was least in the conventional dentifrice group when compared with other groups and this difference was statistically significant (*p*=0.001). At the end of 45 days (T4) and 60 days (T5), the mean GI score was lower in the conventional dentifrice group and herbal dentifrice group when compared with other groups and this difference was statistically significant (*p*=0.001). The mean percentage reduction in GI scores from baseline to 60 days in groups I, II, III, and IV are 29.19%, 22.28%, 32.43%, and 10.33% respectively. Higher reduction in GI scores was seen in Group III followed by Group I at the end of the study period.

**Table 2:**
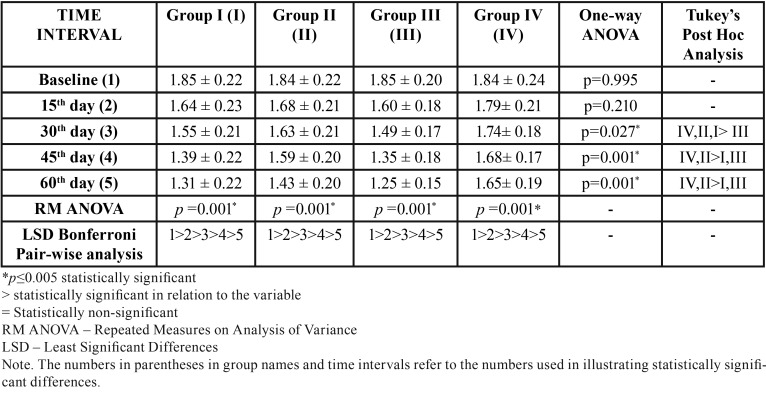
Inter and intra group comparison of mean Gingival Index scores at various time intervals.

**Figure 3:**
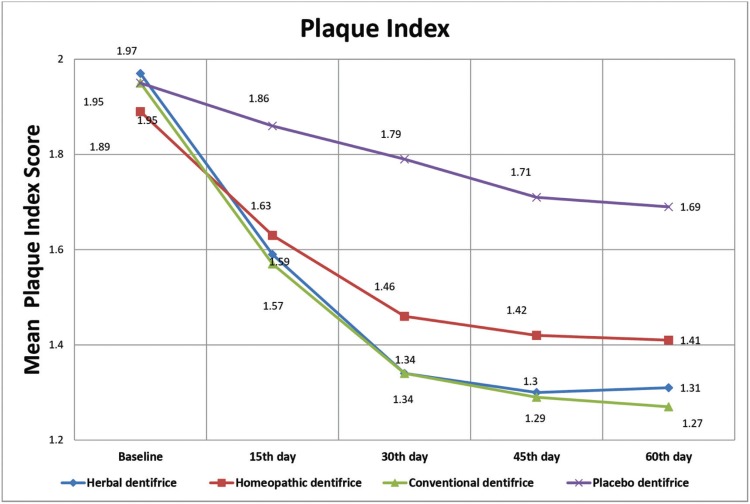
Comparison of the dentifrice groups with respect to mean Plaque Index scores at various time intervals.

Table 3 and Figure 3 outlines the inter-group and intra-group comparison of mean PI scores. The mean Plaque Index (PI) scores in the herbal (Group I), homeopathic (Group II), conventional (Group III) and placebo dentifrice (Group IV) groups were 1.97±0.22, 1.89± 0.28, 1.95 ± 0.32 and 1.95± 0.23respectively at baseline (T1). There was no statistically significant difference between the groups (*p*=0.818). The statistical difference between the groups was non-significant (*p*= 0.318) even at the end of 15 days (T2). At the end of 30 days (T3), 45 days (T4) and 60 days (T5), the mean PI score was least in the conventional and herbal dentifrice groups when compared with other groups and this difference was statistically significant (*p*=0.001). The mean percentage reduction in PI scores from baseline to 60 days in groups I, II, III, and IV are 33.5%, 25.4%, 34.87%, and 13.34% respectively. (Table 4) Higher reduction in PI scores was seen in Group III followed by Group I at the end of the study period.

**Table 3:**
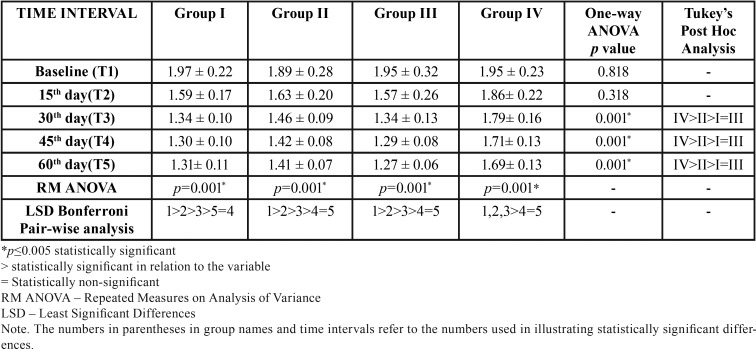
Inter and intra group comparison of mean Plaque Index scores at various time intervals.

**Table 4:**
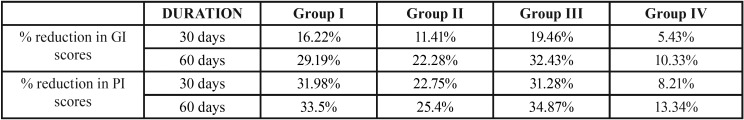
Percentage reduction in mean Gingival Index and Plaque Index scores when compared to baseline and the end of 30 days (T3) and 60 days (T5) of dentifrice usage.

## Discussion

As the soaring popularity of medicines and dentifrices made of natural extracts continues to dominate, dental professionals are in a tight spot to provide information about these products’ safety and efficacy to their patients. This can be difficult, owing to a lack of professional consensus on the subject. To date, an insufficient amount of clinical research on herbal and homeopathic based dentifrices has been reported, in contrast with a plethora of such research for conventional oral care products. A lack of scientific studies on herbal and homeopathic products mushrooming the market in the peer-reviewed dental literature poses a challenge for health care professionals. Hence, this therapeutic clinical trial was conducted with an objective to evaluate and compare the clinical effectiveness of commercially available dentifrices of complementary and alternative medicine systems with that of a conventional dentifrice and a placebo dentifrice on Gingival Index and Plaque Index scores among adults aged 35-44 years.

As no previous research work was conducted to assess and compare the efficacy of CAM dentifrices used in the present study on plaque and gingivitis, an accurate comparison of the present study with other studies may not be possible. However, an attempt was made to compare selected results wherever possible maintaining the validity of comparisons to the possible extent. Also, the results obtained with each dentifrice are ascribed to the active ingredients alone and not to the whole dentifrice, as the study design incorporates suiTable controls (dentifrices) and all the preparations differed from each other only with respect to the active ingredient. The active ingredients in herbal dentifrice were Pomegranate, Neem, Indian gum Arabic tree, Triphala and Miswak. Dhabolkar CS *et al.* reported that Pomegranate was found to improve gingival status due to its profound styptic action (9). Chatterjee *et al.* suggested that the antimicrobial properties of Neem bark extract would improve gingival health and lessen plaque accumulation (10). The stem bark of Indian gum Arabic tree is credited with astringent property and anti-microbial activity which would enhance gingival health (11). Triphala was reported to have a potent anti-oxidant and anti-microbial activity thereby reducing plaque formation (12). Miswak has been found to possess anti-microbial, anti-plaque, anti-inflammatory and astringent activities (13). In the herbal dentifrice group, GI scores and Pl scores had reduced by 29.19% and 33.5% respectively from baseline to the end of 60 days (*p*=0.001). Khairnar M *et al.* demonstrated the anti-inflammatory activity of the herbal dentifrice used in the present study (14) Also Clinical trials performed previously using individual herb either in the form of dentifrice; Zanthoxylumnitidum toothpaste (15) and Acacia Arabica gel (11) or as a mouthwash; Punicagranatum extract, (9) neem based mouth wash (10) and Triphala mouthwash (12) or as Miswak sticks (13) reported a significant reduction in the GI and PI scores when compared to baseline. So the reduction seen in the present study could be attributed to the herbs used in the manufacture of the herbal dentifrice individually and also probable synergistic effect upon combination.

The active ingredients in homeopathic dentifrice were Calendula officinalis, Plantago major, and Hamamelis Virginica. Calendula extract was found to improve gingival inflammatory conditions (16). Plantago major extract is found to have antiseptic properties and interrupts bio-film formation (17). LLL In the homeopathic dentifrice group, GI scores and Pl scores had reduced by 22.28 % and 25.4 % respectively from baseline to the end of 60 days (*p*=0.001). A clinical trial conducted by Khairnar MS *et al.* using Calendula officinalis extract mouthrinse had shown about 47% reduction in GI scores and 50% reduction in PI scores at the end of the study (16). Another study using Plantago major extract reported no effect at all on plaque colonization (17). So, the reduction in GI and PI scores in the present study could be attributed to Calendula extract mainly. Whereas, the combination of calendula extract with Plantago major would have reduced the overall anti-gingivitis effect.

The active ingredients in conventional dentifrice used in the study are Polyvinyl-methyl ether-maleic acid Copolymer (Gantrez) and Triclosan. Existing literature about the dentifrice suggests that it reduces plaque formation, gingivitis, and gingival bleeding owing its ability to alter the quality of plaque bio-film (18,19). In the conventional dentifrice group, GI scores and Pl scores had reduced by 32.43 % and 34.87 % respectively from baseline to the end of 60 days (*p*=0.001). Similar results were obtained in the studies conducted by Garica-Godoy F *et al.* and Lindhe J *et al.* (18,19) These results could be attributed to the plaque inhibitory activity of triclosan wherein it acts on the cyclooxygenase and lipoxygenase pathways of inflammation and inhibits inflammatory mediators (20).

No statistically significant difference was observed in mean GI scores between the four dentifrice groups at baseline and 15 days (*p* > 0.05). At the end of 30 days, the conventional dentifrice was found superior to herbal and homeopathic dentifrices in gingivitis reduction. The anti-gingivitis effect of homeopathic dentifrice was very low and almost the same as placebo. Overall, both the herbal and conventional dentifrices had an equivocal and higher reduction in GI and PI scores by the end of the 60 day study period. Studies conducted by Mullaly BH *et al.*, (21) George J *et al.*, (22) Ozaki F *et al.* (23) and Hebbal M *et al.* (24) reported that the anti-gingivitis and anti-plaque effect of herbal dentifrices is comparable with triclosan based dentifrices whereas studies by Moran J *et al.* (25) and Al-Kholani AI *et al.* (26) established the superiority of conventional dentifrice.

## Conclusions

As plaque is the important etiological factor in initiation and progression of dental caries and periodontal disease, controlling the accumulation of plaque in the oral cavity is of paramount importance to an individual. In order to maintain the optimal oral health and reduce disease burden in the community, public can be encouraged to use herbal dentifrice used in the present study for their routine oral hygiene practice as the observed anti-gingivitis and anti-plaque efficiency was almost similar to established triclosan containing conventional dentifrice. Further long-term studies must be performed to evaluate the anti-gingivitis and anti-plaque effects of many dentifrices of CAM systems available in the market for establishing superiority, as using dentifrices is a life-long event.

## References

[1] Petersen PE (2010). Improvement of global oral health--the leadership role of the World Health Organization. Community Dent Health.

[2] Löe H (2000). Oral hygiene in the prevention of caries and periodontal disease. Int Dent J.

[3] Moran JM (1997). Chemical plaque control--prevention for the masses. Periodontol 2000.

[4] Jayashankar S, Panagoda GJ, Amaratunga EAPD, Perera K, Rajapakse PS (2011). A randomised double-blind placebo-controlled study on the effects of a herbal toothpaste on gingival bleeding, oral hygiene and microbial variables. Ceylon Medical Journal.

[5] Singh A, Purohit B (2011). Tooth brushing, oil pulling and tissue regeneration: A review of holistic approaches to oral health. J Ayurveda Integr Med.

[6] Newadkar UR, Chaudhari L, Khalekar YK (2016). Homeopathy in Dentistry: Is There a Role?. Pharmacognosy Res.

[7] Turesky S, Gilmore ND, Glickman I (1970). Reduced plaque formation by the chloromethyl analogue of victamine C. J Periodontol.

[8] Loe H, Silness J (1963). Periodontal disease in pregnancy. I. Prevalence and Severity. ActaOdontol Scand.

[9] Dabholkar CS, Shah M, Kathariya R, Bajaj M, Doshi Y (2016). Comparative Evaluation of Antimicrobial Activity of Pomegranate-Containing Mouthwash Against Oral-Biofilm Forming Organisms: An Invitro Microbial Study. J Clin Diagn Res.

[10] Chatterjee A, Saluja M, Singh N, Kandwal A (2011). To evaluate the antigingivitis and antipalque effect of an Azadirachtaindica (neem) mouthrinse on plaque induced gingivitis: A double-blind, randomized, controlled trial. J Indian SocPeriodontol.

[11] Pradeep AR, Happy D, Garg G (2010). Short-term clinical effects of commercially available gel containing Acacia arabica: a randomized controlled clinical trial. Aust Dent J.

[12] Naiktari RS, Gaonkar P, Gurav AN, Khiste SV (2014). A randomized clinical trial to evaluate and compare the efficacy of triphala mouthwash with 0.2% chlorhexidine in hospitalized patients with periodontal diseases. J Periodontal Implant Sci.

[13] Sofrata A, Brito F, Al-Otaibi M, Gustafsson A (2011). Short term clinical effect of active and inactive Salvadorapersica miswak on dental plaque and gingivitis. J Ethnopharmacol.

[14] Khairnar MR, Dodamani AS, Karibasappa GN, Naik RG, Deshmukh MA (2017). Efficacy of herbal toothpastes on salivary pH and salivary glucose - A preliminary study. J Ayurveda Integr Med.

[15] Wan HC, Hu DY, Liu HC (2005). Clinical observation of toothpaste containing zanthoxylumnitidum extract on dental plaque and gingivitis. ZhongguoZhong Xi Yi Jie He ZaZhi.

[16] Khairnar MS, Pawar B, Marawar PP, Mani A (2013). Evaluation of Calendula officinalis as an anti-plaque and anti-gingivitis agent. J Indian Soc Periodontol.

[17] Sharma H, Yunus G Y, Mohapatra AK, Kulshrestha R, Agrawal R, Kalra M (2016). Antimicrobial efficacy of three medicinal plants Glycyrrhiza glabra, Ficus religiosa, and Plantago major on inhibiting primary plaque colonizers and periodontal pathogens: An in vitro study. Indian J Dent Res.

[18] Garcia-Godoy F, Garcia-Godoy F, DeVizio W, Volpe AR, Ferlauto RJ, Miller JM (1990). Effect of a triclosan/copolymer/fluoride dentifrice on plaque formation and gingivitis: a 7-month clinical study. Am J Dent.

[19] Lindhe J, Rosling B, Socransky SS, Volpe AR (1993). The effect of a triclosan-containing dentifrice on established plaque and gingivitis. J Clin Periodontol.

[20] Gaffar A, Scherl D, Afflitto J, Coleman EJ (1995). The effect of triclosan on mediators of gingival inflammation. J Clin Periodontol.

[21] Mullally BH, James JA, Coulter WA, Linden GJ (1995). The efficacy of a herbal-based toothpaste on the control of plaque and gingivitis. J ClinPeriodontol.

[22] George J, Hegde S, Rajesh KS, Kumar A (2009). The efficacy of a herbal-based toothpaste in the control of plaque and gingivitis: a clinico-biochemical study. Indian J Dent Res.

[23] Ozaki F, Pannuti CM, Imbronito AV, Pessotti W, Saraiva L, de Freitas NM (2006). Efficacy of a herbal toothpaste on patients with established gingivitis--a randomized controlled trial. Braz Oral Res.

[24] Hebbal M, Ankola AV, Sharma R, Johri S (2012). Effectiveness of herbal and fluoridated toothpaste on plaque and gingival scores among residents of a working women's hostel - a randomised controlled trial. Oral Health Prev Dent.

[25] Moran J, Addy M, Newcombe R (1991). Comparison of an herbal toothpaste with a fluoride toothpaste on plaque and gingivitis. Clin Prev Dent.

[26] Al-Kholani AI (2011). Comparison between the Efficacy of Herbal and Conventional Dentifrices on Established Gingivitis. Dent Res J (Isfahan).

